# Assessment of the predictive accuracy of five *in silico* prediction tools, alone or in combination, and two metaservers to classify long QT syndrome gene mutations

**DOI:** 10.1186/s12881-015-0176-z

**Published:** 2015-05-13

**Authors:** Ivone US Leong, Alexander Stuckey, Daniel Lai, Jonathan R Skinner, Donald R Love

**Affiliations:** Diagnostic Genetics, LabPlus, Auckland City Hospital, Auckland, New Zealand; Bioinformatics Institute, University of Auckland, Auckland, New Zealand; Green Lane Paediatric and Congenital Cardiac Services, Starship Children’s Hospital, Private Bag 92024, Auckland, 1142 New Zealand; Cardiac Inherited Disease Group, Auckland City Hospital, Auckland, New Zealand; Department of Child Health, University of Auckland, Auckland, New Zealand

**Keywords:** Long QT syndrome, Genetics, Ion channels, *In silico* prediction tools

## Abstract

**Background:**

Long QT syndrome (LQTS) is an autosomal dominant condition predisposing to sudden death from malignant arrhythmia. Genetic testing identifies many missense single nucleotide variants of uncertain pathogenicity. Establishing genetic pathogenicity is an essential prerequisite to family cascade screening. Many laboratories use *in silico* prediction tools, either alone or in combination, or metaservers, in order to predict pathogenicity; however, their accuracy in the context of LQTS is unknown. We evaluated the accuracy of five *in silico* programs and two metaservers in the analysis of LQTS 1–3 gene variants.

**Methods:**

The *in silico* tools SIFT, PolyPhen-2, PROVEAN, SNPs&GO and SNAP, either alone or in all possible combinations, and the metaservers Meta-SNP and PredictSNP, were tested on 312 *KCNQ1*, *KCNH2* and *SCN5A* gene variants that have previously been characterised by either *in vitro* or co-segregation studies as either “pathogenic” (283) or “benign” (29). The accuracy, sensitivity, specificity and Matthews Correlation Coefficient (MCC) were calculated to determine the best combination of *in silico* tools for each LQTS gene, and when all genes are combined.

**Results:**

The best combination of *in silico* tools for *KCNQ1* is PROVEAN, SNPs&GO and SIFT (accuracy 92.7%, sensitivity 93.1%, specificity 100% and MCC 0.70). The best combination of *in silico* tools for *KCNH2* is SIFT and PROVEAN or PROVEAN, SNPs&GO and SIFT. Both combinations have the same scores for accuracy (91.1%), sensitivity (91.5%), specificity (87.5%) and MCC (0.62). In the case of *SCN5A,* SNAP and PROVEAN provided the best combination (accuracy 81.4%, sensitivity 86.9%, specificity 50.0%, and MCC 0.32). When all three LQT genes are combined, SIFT, PROVEAN and SNAP is the combination with the best performance (accuracy 82.7%, sensitivity 83.0%, specificity 80.0%, and MCC 0.44). Both metaservers performed better than the single *in silico* tools; however, they did not perform better than the best performing combination of *in silico* tools.

**Conclusions:**

The combination of *in silico* tools with the best performance is gene-dependent. The *in silico* tools reported here may have some value in assessing variants in the *KCNQ1* and *KCNH2* genes, but caution should be taken when the analysis is applied to *SCN5A* gene variants.

**Electronic supplementary material:**

The online version of this article (doi:10.1186/s12881-015-0176-z) contains supplementary material, which is available to authorized users.

## Background

Long QT syndrome (LQTS) is a heritable cardiac disorder characterised by a prolonged QT-interval detected on an electrocardiogram (ECG), episodes of syncope, and the risk of sudden death. The estimated number of affected people is 1 in 2,500 [1,2], and 13 causative genes have been identified. The majority of these genes encode for the cardiac ion channels (potassium, sodium and calcium). Loss-of-function mutations in the *KCNQ1* and *KCNH2* genes (LQT1 and LQT2, respectively) account for ~65% of all LQTS cases, and gain-of-function mutations in the *SCN5A* gene (LQT3) account for 10% of all LQTS cases [3,4]. Genotype-phenotype correlation studies have established genotype-specific ECG patterns, arrhythmia triggers and outcomes [5,6]. Genetic diagnosis permits better risk stratification, clinical management and cascade family screening [7].

As genetic testing becomes more widely available, an increasing number of variants of unknown/uncertain significance are being discovered. Before family cascade screening can be undertaken, it is essential to be confident of the pathogenicity of the variant. While nonsense and frameshift mutations, which cause premature termination of protein production, are usually pathogenic, missense mutations are very commonly benign. The interpretation of novel single nucleotide variants (SNVs) is difficult. SNVs represent ~75% of clinically positive LQTS test results [8]. Approximately 1 in 25 healthy individuals are expected to have a rare, benign variant in one of the three major LQTS genes [9,10]. The best methods to assess the pathogenicity of novel mutations are to undertake phenotype-genotype family co-segregation studies, and functional/biophysical studies in an *in vitro* system or animal models. However, many families are too small, and the latter two methods are expensive and time-consuming and unavailable to most diagnostic services. With the growing number of unclassified SNVs, many diagnostic laboratories use *in silico* missense mutation prediction tools to determine whether a novel SNV is pathogenic in relation to the evolutionary conservation of specific amino acids, as well as protein structure and function.

The prediction tools can be broadly divided into three categories: sequence and evolutionary conservation-based methods, protein sequence and structure-based methods, and supervised learning methods (refer to [3,11-13] for reviews of the different categories). The sequence and evolutionary conservation-based methods assess the pathogenicity of a mutation based on the conservation of a particular amino acid across different species [14-16]. Protein sequence and structure-based methods assess SNVs based on their location in the protein structure and how they may impact/disrupt the overall protein [17]. The supervised learning methods are trained on large defined datasets so that they can “learn” to distinguish pathogenic mutations from benign variants [15,18,19].

Several studies have investigated the use of multiple *in silico* prediction tools to assess the predictive accuracy of these tools [20-28]. There are also consensus programs (metservers) that combine the output from several *in silico* prediction tools and produce a single consensus outcome, all of which have been reported to offer improved performance over individual tools [29-31]. However, no study has investigated the combination(s) of individual *in silico* prediction tools with the best performance for LQTS genes or whether the use of metaservers is better.

The aim of the study described here was to assess the predictive accuracy of five *in silico* programs (SIFT, PolyPhen-2, PROVEAN, SNPs&GO and SNAP), alone or in combination, and two metaservers (Meta-SNP and PredictSNP) in evaluating SNVs in the three major LQTS genes (*KCNQ1*, *KCNH2* and *SCN5A*). All mutations reported in the Inherited Arrhythmia Database [32] for these genes were analysed to determine the combination of the *in silico* programs with the best performance, and how the combinations compared to the use of the metaservers.

## Methods

All LQTS gene SNVs (both deleterious, polymorphisms and rare SNVs) were collated from the Inherited Arrhythmia Database (http://www.fsm.it/cardmoc/ last accessed October 2014), Kapplinger et al. [33], Giudicessi et al. [5] and the LQTS gene LOVD database [34]. Only SNVs that caused missense amino acid changes were considered for analysis. For this study the SNVs were divided into two groups: pathogenic and benign. To be considered for the pathogenic group, SNVs must either be functionally characterised by *in vitro* and/or have undergone co-segregation studies to prove they are pathogenic. In the case of the benign group, SNVs must either be functionally characterised by *in vitro* studies and/or must have an allele frequency of greater than 1%. SNVs in the *SCN5A* gene reported by Kapplinger et al. [33] were found in either LQTS and/or Brugada syndrome patients. All the mutations that were analysed and their respective results are shown in Additional file 1: Data tables, and their locations in respect of the different protein regions (transmembrane domains, pore regions, etc.) are shown in Additional file 2: Figures S1–S3.

Five *in silico* missense mutation prediction tools and two metaservers, listed in Table 1, were used to analyse all the LQTS genes SNVs and the exact methodology and algorithms used by each of these have been described previously according to the references given here: PolyPhen-2 [17], SIFT [14], PROVEAN [16], SNPs&GO [18,35], SNAP [19], Meta-SNP [30] and PredictSNP [31].

**Table 1:** ***In silico*** **prediction tools and metaservers used in the current study**

**Program**	**Type**	**Incorporated programs (Metaserver only)**	**Type (Metaserver only)**	**URL**
PolyPhen-2, version 2.2.2 [17]	Protein sequence and structure			http://genetics.bwh.harvard.edu/pph2/
SNPs&GO [18]	Supervised learning (support vector machine)			http://snps.biofold.org/snps-and-go/snps-and-go.html
SIFT, version 5.2.0 [14,15]	Sequence and evolutionary conservation			http://siftdna.org/www/Extended_SIFT_chr_coords_submit.html
PROVEAN, version 1.1 [16]	Sequence and evolutionary conservation			http://provean.jcvi.org/index.php
SNAP [19]	Supervised learning (neural networks)			https://www.rostlab.org/services/SNAP/
Meta-SNP [30]	Metaserver	PANTHER	Sequence and evolutionary conservation	http://snps.biofold.org/meta-snp/
		PhD-SNP	Supervised-learning (support vector machines)	
		SIFT	Sequence and evolutionary conservation	
		SNAP	Supervised learning (neural networks)	
PredictSNP [31]	Metaserver	MAPP	Sequence and evolutionary conservation	http://loschmidt.chemi.muni.cz/predictsnp/
		PhD-SNP	Supervised-learning (support vector machines)	
		PolyPhen-1	Protein sequence and structure	
		PolyPhen-2	Protein sequence and structure	
		SIFT	Sequence and evolutionary conservation	
		SNAP	Supervised learning (neural networks)	

PolyPhen-2 uses annotated UniProt entries to predict if a missense mutation is situated in a structurally important/functional site in the protein using a naïve Bayesian approach [17]. Therefore, PolyPhen-2 could belong to the supervised-learning method of *in silico* analysis [13]. LQTS SNVs were analysed by accessing the web-based method PolyPhen-2, using default settings [17]. PolyPhen-2 classified each variant as “Probably damaging”, “Possibly damaging”, or “Benign”. For the purposes of this study, SNVs assigned as “Probably damaging” or “Possibly damaging” were classified as “damaging” for downstream analysis.

SNPs&GO incorporates sequence information, evolutionary information, and information from the GO (Gene Ontology) database [18]. The default settings were used. SNPs&GO classified each mutation variant as either “Neutral” or “Disease” [15,18].

SIFT uses sequence homology from multiple sequence alignments to predict the pathogenicity of a mutation [14,15]. SIFT non-synonymous single nucleotide variants (genome-scale) was used. The chromosomal location, genomic coordinate, transcript orientation and base-pair change of each SNVs were required for the SIFT nonsynonymous single nucleotide variants (genome-scale) input format. The web-based Variant Effect Predictor – Ensembl (http://asia.ensembl.org/info/docs/tools/vep/index.html) was used to generate the required information. The default settings were used for this study. SIFT classified each variant as either “Tolerated” or “Damaging”.

The web-based PROVEAN Protein Batch Human was used [16]. The PROVEAN algorithm classified each SNV as either “Neutral” or “Deleterious”.

SNAP makes predictions based on protein secondary structure, solvent accessibility and the conservation of the amino acid of interest in a protein [19]. The default settings were used. SNAP classified each mutation as either “Neutral” or “Non-neutral” [19].

Meta-SNP [30] and PredictSNP [31] are metaservers that combine the predicted outcomes from several *in silico* tools to form a consensus prediction for a given SNV. Meta-SNP uses a random forest approach to integrate the predictions from *in silico* tools [30] (these are listed in Table 1). Each mutation is classified as either “Disease” or “Neutral” [30]. PredictSNP is a consensus classifier that integrates the results from six *in silico* prediction tools (listed in Table 1) as well as experimental annotations from Protein Mutant Database and UniProt [31].

The raw data output of all LQTS gene SNVs can be found in the Additional file 3: Raw Data. The functional predictions of all LQTS gene SNVs from all five *in silico* tools and two metaservers were collated in Excel spreadsheets. The location of each LQTS gene mutation in the context of protein structure, as well as information about whether the SNVs were functionally characterised are shown in Additional file 1: Data tables and Additional file 2: Figures S1–S3.

The data output were initially compared with the functional studies’ results to determine the accuracy of the programs. SNVs that were not functionally characterised but were proven to be pathogenic through co-segregation studies, or had an allelic frequency of greater than 1%, were assumed to be pathogenic or benign, respectively. These results were subcategorised into four groups: true positives (TP, correct predictions for deleterious mutations), true negatives (TN, correct predictions for neutral mutations), false positives (FP, incorrect predictions for neutral mutations) and false negatives (FN, incorrect predictions for deleterious mutations). The sensitivity (true positive rate) and specificity (true negative rate) for each *in silico* tool were determined using the four different categories. Sensitivity was defined as the probability of identifying true deleterious mutations, and this was calculated by [TP/(TP + FN)] x 100 [36]. Specificity was defined as the probability of identifying true neutral mutations, and this was calculated by [TN/(TN + FP)] x 100 [36]. The Matthews Correlation Coefficient (MCC) was also calculated for each category using the following equation (TP x TN) – (FP x FN)/sqrt((TP + FP)(TP + FN)(TN + FP)(TN + FN)) [37]. The MCC measures how the predictions correlate with the real target values, and the scores range from +1 (always correct) to −1 (always false), and 0 represents a completely random prediction [36]. An MCC score of more than 0.5 was considered acceptable as this corresponds to more than 75% accuracy in balanced data [38].

Receiver operating characteristic (ROC) curves and the area under the ROC curve (AUC) [39] for each of the single *in silico* tools and the two metaservers were calculated using the pROC package in R [40]. ROC curve plots sensitivity against 1-specificity, which depicts the relative tradeoffs between the true positives and false positives [39]. The probability scores of each *in silico* prediction tool were used to calculate the curves and AUC. An AUC of 1 represents a perfect prediction, an AUC of 0.5 relates to predictions that are made by “pure chance”, and an AUC less than 0.5 shows the predictions are wrong.

The sensitivity percentage represents how well the *in silico* tools and metaservers correctly predict pathogenicity, and the specificity percentage represents how well they correctly predict non-pathogenic outcomes [36]. MCC, ROC curve and AUC were a more balanced overall evaluation of the ability of the prediction tools to correctly classify the SNVs compared to just analysing the accuracy. ROC curves do not directly indicate the performance of a method, but only shows the method’s ranking potential for its overall performance [38].

The data output for each *in silico* tool were then analysed in combinations of two, three, four or all five *in silico* tools, and the accuracy of the predictions, sensitivity, specificity and MCC were determined for each combination. The conditions that an SNV must meet in order to be categorised as “Tolerated” or “Damaging” in the case of being assessed by two, three, four or all five *in silico* missense prediction tools are shown in Table 2. The AUC scores were used as an indicator as to which *in silico* tools performed best for a particular LQTS gene.

**Table 2:** **Conditions for SNV data output from two, three, four and all** ***in silico*** **missense prediction tools in order to be considered tolerated and damaging**

**Number of** ***in silico*** **prediction tools**	**SNV considered as Tolerated**	**SNV considered as Damaging**
Two tools	Unanimous neutral/tolerated/benign	Unanimous damaging/disease/deleterious/non-neutral
		One output is damaging/disease/deleterious/non-neutral
Three tools	Unanimous neutral/tolerated/benign	Unanimous damaging/disease/deleterious/non-neutral
	Two outputs are neutral/tolerated/benign	Two outputs are damaging/disease/deleterious/non-neutral
Four tools	Unanimous neutral/tolerated/benign	Unanimous damaging/disease/deleterious/non-neutral
	Three outputs are neutral/tolerate/benign	Two or more outputs are damaging/disease/deleterious/non-neutral
All tools	Unanimous neutral/tolerated/benign	Unanimous damaging/disease/deleterious/non-neutral
	Three outputs are neutral/tolerated/benign	Three or more outputs are damaging/disease/deleterious/non-neutral

The differences between single tools and differing combinations of tools in accuracy was calculated using the Kruskal-Wallis test [41]. The best performing *in silico* tool/combination of tools were chosen based on their MCC scores, which is a more balanced approach to investigate performance because it is less sensitive to different numbers of pathogenic and benign SNVs for each gene [36].

## Results

**Figure 1:**
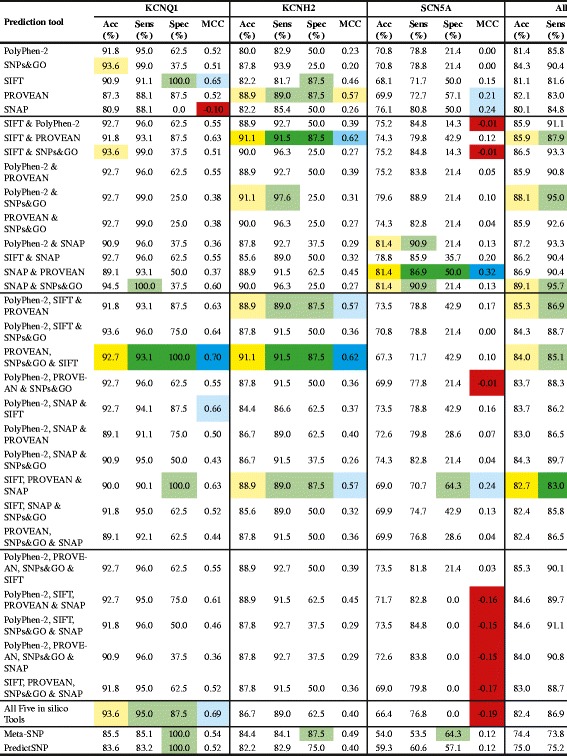
The accuracy (acc), sensitivity (sens), specificity (spec) and MCC scores of all the different combinations of *in silico* prediction tools and metaservers for variants in the *KCNQ1, KCNH2, SCN5A* genes, and all genes combined. The sensitivity and specificity percentages highlighted in light green represent the combination of *in silico* tools and metaservers with high sensitivity or specificity percentages; dark green represents the combination of *in silico* tools or metaservers with the best performance; light blue represents the combination of *in silico* tools and metaservers with high MCC scores, and dark blue represents the combination of *in silico* tools or metaservers with the best performance; light orange represents the combination of *in silico* tools and metaservers with high accuracy percentages; yellow represents the combination of *in silico* tools or metaservers with the best performance; red represents the combination of *in silico* tools and metaservers with MCC scores below 0.

**Figure 2:**
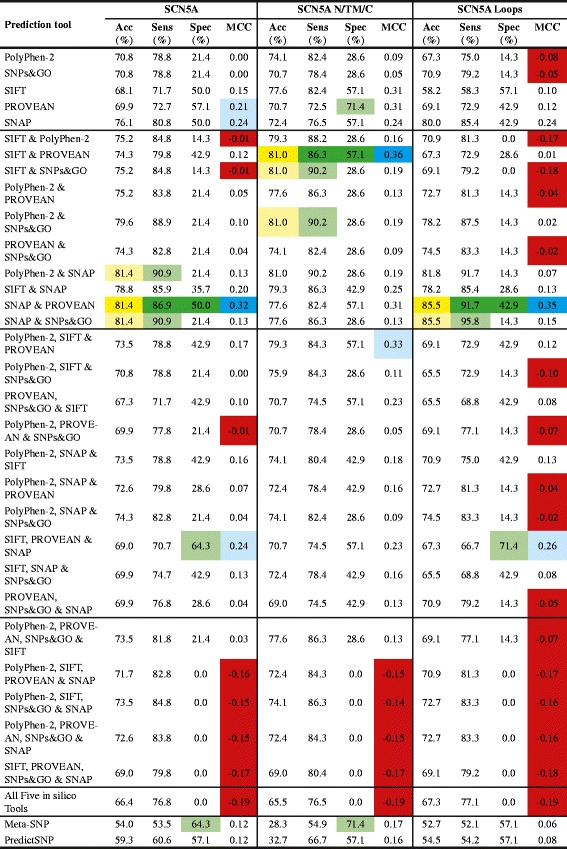
The accuracy (acc), sensitivity (sens), specificity (spec) and MCC scores of all the different combinations of *in silico* prediction tools and metaservers for the whole of SCN5A, only the amino-/carboxyl-terminus and transmembrane domain (N/TM/C) of SCN5A, and only the loop regions of SCN5A. The sensitivity and specificity percentages highlighted in light green represent the combination of *in silico* tools and metaservers with high sensitivity or specificity percentages; dark green represents the combination of *in silico* tools or metaservers with the best performance; light blue represents the combination of *in silico* tools and metaservers with high MCC scores, and dark blue represents the combination of *in silico* tools or metaservers with the best performance; light orange represents the combination of *in silico* tools and metaservers with high accuracy percentages; yellow represents the combination of *in silico* tools or metaservers with the best performance; red represents the combination of *in silico* tools and metaservers with MCC scores below 0.

A total of 312 missense SNVs (including pathogenic and benign variants, see Table 3) in three LQTS genes were analysed using five different *in silico* missense prediction tools and two metaservers. To verify that the combined *in silico* tools have different decision cutoffs, pairwise correlations of the *in silico* prediction tools were calculated to ensure no strong correlation between any pair of tools would affect the combined results (Additional file 4: Tables S1–S4). ROC curves were calculated for each individual *in silico* prediction tools and metaservers (Table 4), and the possible outcomes for the five *in silico* tools and metaservers are shown in Figures 1 and 2.

**Table 3:** **The total number of functionally characterised** ***KCNQ1*** **,** ***KCNH2*** **and** ***SCN5A*** **gene variants investigated in this study**

**Gene**	**Pathogenic**	**Benign**	**Total**
*KCNQ1*	101	8	109
*KCNH2*	82	8	90
*SCN5A*	99	14	113
Total			312

**Table 4:** **Area under the curve (AUC) calculated from Receiver operating curves for each of the** ***in silico*** **prediction tools alone using their respective probabilities/scores for** ***KCNQ1*** **,** ***KCNH2*** **,** ***SCN5A*** **and all genes combined**

**Programs**	***KCNQ1***		***KCNH2***		***SCN5A***		**All genes**	
	**AUC**	**CI**	**AUC**	**CI**	**AUC**	**CI**	**AUC**	**CI**
PolyPhen-2	0.942	(0.877-1.000)	0.774	(0.624-0.924)	0.626	(0.485-0.767)	0.769	(0.688-0.850)
SNPs&GO	0.933	(0.882-0.984)	0.864	(0.752-0.976)	0.666	(0.528-0.803)	0.781	(0.714-0.849)
SIFT	0.834	(0.687-0.982)	0.819	(0.729-0.910)	0.643	(0.450-0.836)	0.715	(0.602-0.828)
PROVEAN	0.943	(0.891-0.995)	0.904	(0.840-0.968)	0.631	(0.456-0.807)	0.786	(0.689-0.883)
SNAP	0.689	(0.500-0.877)	0.397	(0.194-0.600)	0.590	(0.421-0.758)	0.627	(0.522-0.732)
Meta-SNP	0.959	(0.918-0.999)	0.905	(0.826-0.984)	0.639	(0.506-0.772)	0.839	(0.781-0.897)
PredictSNP	0.713	(0.571-0.856)	0.549	(0.373-0.725)	0.555	(0.397-0.714)	0.603	(0.513-0.693)

### KCNQ1

**Figure 3:**
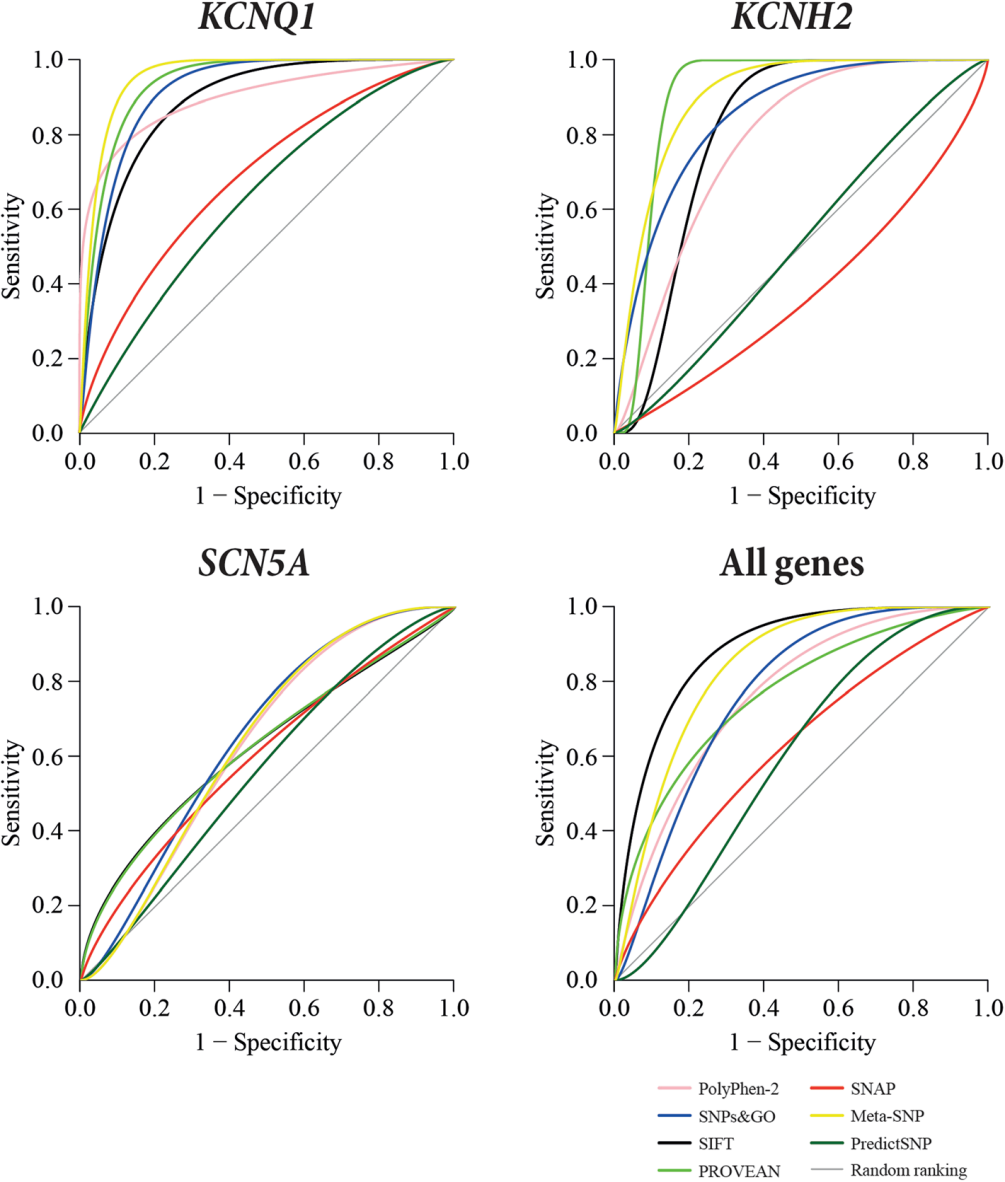
Receiver operating characteristic (ROC) curves for *KCNQ1*, *KCNH2*, *SCN5A* and all genes combined for each of the single *in silico* prediction tools and the two metaservers.

The ROC curves of the individual *in silico* prediction tools and metaservers showed that Meta-SNP, PROVEAN, SNPs&GO and SIFT were the best tools for *KCNQ1* gene predictions (Figure 3). The AUC scores showed that Meta-SNP, PROVEAN, PolyPhen-2 and SNPs&GO were the best tools (Table 4), while MCC scores ranked SIFT, Meta-SNP, PolyPhen-2, PROVEAN and PredictSNP as the best tools (Figure 1) for *KCNQ1* gene predictions.

There were no significant differences between the accuracy of the different combinations of *in silico* tools and the metaservers alone. However, based on the MCC scores, the best combination of *in silico* tools for *KCNQ1* was PROVEAN, SNPs&GO and SIFT (MCC score of 0.70; Figure 1). This combination had an accuracy of 92.7%, sensitivity of 93.1% and specificity of 100% (Figure 1). Despite not having the highest accuracy (94.5% achieved by SNAP and SNPs&GO), the combination of PROVEAN, SNPs&GO and SIFT was able to correctly classify both pathogenic and benign SNVs equally well (Figure 1). When all five *in silico* tools were used, the combined results were almost as good with an MCC score of 0.69 (Figure 1). The two combinations of tools also surpassed the performance of both metaservers in terms of accuracy, sensitivity and MCC scores.

### KCNH2

According to the ROC curves, PROVEAN, Meta-SNP, SIFT and SNPs&GO were the best tools (Figure 3), and the AUC scores ranked Meta-SNP, PROVEAN, SNPs&GO and SIFT as the best tools for *KCNH2* gene predictions (Table 4). PROVEAN, Meta-SNP and SIFT had the highest MCC scores (Figure 1).

Like *KCNQ1*, there were no significant differences in the accuracy of the different combinations of *in silico* tools and the metaservers alone. Based on the MCC scores, SIFT and PROVEAN, and PROVEAN, SNPs&GO and SIFT were the best combinations for *KCNH2* gene predictions (Figure 1). Both combinations had an accuracy of 91.1%, with sensitivity of 91.5%, specificity of 87.5%, and an MCC score of 0.62 (Figure 1). PROVEAN-alone; PolyPhen-2, SIFT and PROVEAN and SIFT, PROVEAN and SNAP were almost as good (MCC of 0.57; Figure 1). The two metaservers did not perform as well compared to the two best combinations of *in silico* tools.

### SCN5A

SNVs in the *SCN5A* gene cause either LQTS and/or Brugada syndrome (BrS) and the analysis was performed on the group of SNVs without separating them by phenotype. The combination of *in silico* tools with the best performance, based on the MCC scores, remains the same for the *SCN5A* gene regardless of whether BrS variants were included or not (Additional file 4). No single predictive tool appeared to work well for the *SCN5A* gene (Figure 3), and the AUC scores ranked SNPs&GO, SIFT, Meta-SNP and PolyPhen-2 as the best programs (Table 4); however, the scores suggested that the chances of the correct prediction being made was a little better than “pure chance”. This was also reflected by the MCC scores (Figure 1).

None of the combinations of *in silico* tools performed as well for the *SCN5A* gene as for *KCNQ1* and *KCNH2* genes (Figure 1). There were no significant differences between the different combination of *in silico* tools regarding accuracy. The overall MCC scores were low, with SNAP and PROVEAN giving the highest score of 0.32. For this combination, the accuracy was 81.4%, with sensitivity of 86.9% and specificity of 50.0% (Figure 1). Both metaservers performed poorly.

Due to the poor performance of the *in silico* tools on SNVs in the *SCN5A* gene as a whole, the analysis of the SNVs was separated into two groups: those that lay in the amino-/carboxyl-terminus and all transmembrane domains, and those that lay in all the loop regions of SCN5A. The MCC scores for all *in silico* tools and metaservers tested improved when only the amino-/carboxyl-terminus and all transmembrane domains were considered; however, the scores were still very low compared to both the *KCNQ1* and *KCNH2* gene results (Figure 2). The combination with the best performance for the amino-/carboxyl-terminus and all transmembrane domains of SCN5A was SIFT and PROVEAN (MCC score 0.36; Figure 2). The analysis of variants in the loop regions was very poor, with MCC scores ranging from −0.50 – 0.35 (Figure 2). SNAP and PROVEAN is the best *in silico* tool combination for analysing SNVs in the SCN5A loop regions (MCC score 0.35; Figure 2). Both metaservers performed poorly for these regions (Figure 2). As these results are comparable to the analysis of SCN5A as a whole, it was decided that the combination of tools with the best performance will be based on that analysis.

### All genes

According to the ROC curves SIFT, Meta-SNP, SNPs&GO and PolyPhen-2 were the top four single programs when all three LQTS genes’ SNVs were combined (Figure 3). The AUC scores ranked Meta-SNP, PROVEAN, SNPs&GO and PolyPhen-2 as the best tools for all gene predictions (Table 4). The MCC scores ranked SIFT, PROVEAN, Meta-SNP and PredictSNP as the best tools (Figure 1).

There were no significant differences between the accuracy of the different combinations of *in silico* tools. Based on MCC scores the combination of *in silico* tools with the best performance was SIFT, PROVEAN and SNAP (Figures 1 and 3). This combination had an accuracy of 82.7%, with sensitivity of 83.0%, specificity of 80.0% and MCC of 0.44 (Figure 1). SIFT and PROVEAN, as well as PolyPhen-2, SIFT and PROVEAN, and PROVEAN, SNPs&GO and SIFT, performed almost as well (MCC score 0.43; Figure 1). These three combinations of *in silico* tools had higher accuracy and sensitivity but lower specificity compared to SIFT, PROVEAN and SNAP (Figure 1). The metaservers did not perform as well as the combination of *in silico* tools with the best performance in terms of accuracy and sensitivity (Figure 1).

## Discussion

The current study investigated the combination of *in silico* prediction tools with the best performance for LQT 1–3 genes. The analysis was restricted to mutations in these genes as they account for approximately 70%-75% of congenital LQTS cases, and the remaining genes only make up ~5% of cases [42]. For the minor genes, very few SNVs (both pathogenic and benign) fit the strict criteria set for the current study and therefore these genes were not analysed. In the case of *SCN5A*, both LQTS and BrS SNVs were analysed together. This was done as the distinction between both LQT3 and Brugada syndrome is not clear cut as some mutations in the *SCN5A* gene are associated with both diseases [43-46]; however, the combination of *in silico* tools with the best performance remained the same regardless of whether the LQTS and BrS mutations were separated or not.

PolyPhen-2, SIFT, PROVEAN and SNPs&GO were chosen as they are routinely used in the author’s diagnostic laboratory. Both PolyPhen-2 and SIFT are the most common prediction tools used in diagnostic laboratories. SNAP was chosen as another supervised-learning based tool that incorporates a wider range of features (evolutionary information, structural features and protein annotation information) [47], and it uses a different learning algorithm than SNPs&GO. Both metaservers (Meta-SNP and PredictSNP) were chosen as they incorporate the results from a good selection of *in silico* prediction tools that span different types of methods.

The approach that was used to categorise variants as “pathogenic” or “benign” based on combined results from the five *in silico* prediction tools as shown in Table 2. This approach was taken to ensure that all likely pathogenic SNVs would not be missed and that benign SNVs were correctly called. “Over-calling” pathogenic SNVs may occur when an even number of *in silico* tools are used as the conditions set out for this study “call” an SNV with equal numbers of “pathogenic” and “benign” results as “pathogenic”. For *KCNQ1*, 4%-15% of SNVs; *KCNH2*, 5%-21% of SNVs; and *SCN5A*, 8%-24% of SNVs fall into this category. However, these conditions ensured that the classification of “benign” SNVs is more stringent, and so would minimise the chance of a possible pathogenic SNVs being classified as “benign” and therefore dismissed.

*In silico* tools that correctly predict pathogenic variants do not necessarily perform well for benign predictions. The combinations that were chosen for all three genes were based on how well the prediction tools were able to identify *both* pathogenic and benign SNVs, thereby reducing the number of false positive and false negative calls. The tools chosen for each gene do not necessarily yield the highest accuracy. In the clinical setting it may in fact be preferable to use an *in silico* tool which is likely to under-call the likelihood of malignancy in favour of improved specificity. This will mean that in a family cascade, fewer people will be erroneously labelled as having the disease on the basis of a genetic test result. In the case of calling a truly pathogenic variant as benign, the clinician must use clinical evaluation of family members in order to reveal the truth in a segregation study. In the current study, the optimal number of *in silico* tools for *KNCQ1, KCNH2,* and when all genes are considered together, is three. Only *SCN5A* requires two *in silico* tools to make the best predictions. Therefore, the “over-calling” issue should be minimised.

The current study also highlights the need to systematically test which combinations of *in silico* tools perform the best for a given gene, and not assume that a large number of programs will provide the best prediction outcomes. The results from these *in silico* methods disagree frequently due to the different algorithms they are based on [21,23]. When considering the predictions for pathogenic *KCNQ1* SNVs by all five *in silico* tools, 82 SNVs (81%) had concordant results; however, when only considering *KCNQ1*’s combination of tools with the best performance (PROVEAN, SNPs&GO and SIFT), an additional six SNVs were agreed upon (88 SNVs, 88%; Additional file 2). In the case of benign *KCNQ1* SNVs, there were no improvements when considering only the results of the tools with the best performance compared to considering all five tools (two SNVs, 25%; Additional file 2). All five tools agreed for 58 *KCNH2* pathogenic SNVs (71%), and this increased to 68 SNVs (83%) when only considering the results from the combination of tools with the best performance. There were three benign SNVs (37.5%) that had the same predictions for all five tools and this increased by one SNV (4, 50%; Additional file 2) when considering the tools with the best performance. In the case of pathogenic *SCN5A* gene mutations, 61 SNVs (62%) had the same predictions for all five *in silico* tools and this increased to 79 SNVs (80%) when only considering PROVEAN and SNAP (Additional file 2). Five benign *SCN5A* SNVs (36%) had the same predictions for all five tools and this increased by two SNVs (7, 50%) when only considering PROVEAN and SNAP (Additional file 2). When considering the predictions for all pathogenic SNVs, 201 SNVs (71%) had the same predictions for all five *in silico* tools; however, when considering the results from PROVEAN, SIFT and SNAP, this increased by 26 SNVs (227, 81%; Additional file 2). For all benign mutations, only 10 SNVs (33%) had the same predictions from all five tools, and this increased by four SNVs (14, 47%) when only PROVEAN, SIFT and SNAP were considered (Additional file 2).

Both *KCNQ1* and *KCNH2* encode for cardiac potassium channels and the structure of these two proteins are very similar. This could be the reason why the combination of *in silico* prediction tools with the best performance are the same for these two genes (PROVEAN, SNPs&GO and SIFT), with *KCNH2* having SIFT and PROVEAN as an additional combination. The reason for both SIFT and PROVEAN working well together could be because they belong to the sequence and evolutionary conservation-based evaluation method, which relies solely on evolutionary sequence conservation information and does not take into account protein structural information (unlike PolyPhen-2) [14-16]. Studies conducted by *Chan et al*. [20] showed that methods based on evolutionary sequence conservation had high predictive values regardless of whether protein information is used. The addition of SNPs&GO to SIFT and PROVEAN could be because SNPs&GO uses evolutionary derived information [18], which is similar to SIFT and PROVEAN, and the inclusion of the information from the Gene Ontology database makes this combination best suited for the *KCNQ1* and *KCNH2* genes.

No prediction tools are considered suitable for analysing variants in the *SCN5A* gene from this study. The results for tools that incorporate protein structure and function into their algorithms (PolyPhen-2, SNPs&GO and SNAP) had low specificity suggesting that functional and structural information hampered predictions for variants in the *SCN5A* gene. This may be due to the two different *SCN5A* isoforms present in the normal human heart. The isoforms differ by only one amino acid (NP_000326 has glutamine-1077 deleted compared to NP_932173) [48]. The transcript encoding for NP_000326 represents 65% of the *SCN5A* gene in the normal heart [48], and depending on which isoform the SNV is present in, the effect of the mutation may differ [49]. A study investigating the functional characteristics of eight common *SCN5A* gene polymorphisms found five of the eight polymorphisms were similar to the unaffected SCN5A protein in the NP_000326 isoform, and only three of the eight were similar to the unaffected SCN5A protein in the NP_932173 isoform [49]. The polymorphisms that affected the function of the SCN5A protein, regardless of which isoform they were present in, affected the protein in different ways [49]. These results could account for the large number of polymorphisms characterised as damaging in this study (14 of 18 polymorphisms were classified as damaging; Additional file 1: Data Tables); of relevance here is the need to specify the protein isoform in some of the *in silico* programs.

Another confounding factor in the *in silico* analysis may lie in the fact that some *SCN5A* SNVs can cause both LQTS and BrS. Gain-of-function mutations are associated with BrS and loss-of-function mutations are associated with LQTS [50]. Flanagan et al., found that both SIFT and PolyPhen-2 had more success predicting loss-of-function compared to gain-of-function mutations [22] and this could also be the case with the other three *in silico* tools used here, hence the low MCC and AUC scores.

The protein context (isoform) of SNVs in SCN5A highlights an important issue when reporting results for diagnostic tests using *in silico* prediction tools. These tools make predictions based only on amino acid sequence and protein structure information, and very little information about protein function. Caution should be used when making clinical diagnosis based solely on predictive results. As demonstrated by the two isoforms of SCN5A, a single amino acid difference can have a significant effect in terms of protein function and interactions between the ion channels and their accessory proteins. The ideal prediction tool should incorporate information of not just the amino acid sequence and protein structure, but also the protein’s function and interaction with other proteins. While some of the latter aspects are included in the SNPs&GO and SNAP programs, more research is required to resolve protein function and interactions in order for these to be incorporated into prediction tools. Therefore, *in silico* predictions should act as an indicator of whether a variant of unknown significance is pathogenic or benign, and if functional studies are available, clinical information should be used to characterise the variant.

Compared to the different combinations of *in silico* tools, the metaservers were not significantly better despite their claims of improved performance over individual integrated tools [30,31]. Both Meta-SNP’s and PredictSNP’s performance were comparable to many of the different combinations of *in silico* prediction tools, with Meta-SNP performing slightly better. The metaservers performed better than individual *in silico* tools; however, compared to the combination of tools with the best performance the metaservers’ accuracy, sensitivity and MCC scores were not as good. Despite this the metaservers had high specificity compared to some of the *in silico* tools.

A major limitation of this study is the low number of benign SNVs for all three LQTS genes. Attempts to address this deficiency by including polymorphisms that have not been functionally characterised only led to a marginal increase in benign SNVs. Therefore, the analysis of the ability of the *in silico* tools to correctly predict an SNV to be benign may not be as reliable as the analysis of the tools’ ability to correctly predict an SNV to be pathogenic.

Another limitation is that in the current study only SIFT, PolyPhen-2, PROVEAN, SNPs&GO, SNAP, Meta-SNP and PredictSNP were investigated in the prediction of SNVs for LQT 1–3 genes; however, it is not to say that these are the only programs that are effective for these genes. For both *KCNQ1* and *KCNH2*, sequence and evolutionary conservation-based *in silico* prediction tools appear to work best as demonstrated by the success of PROVEAN, SIFT and SNPs&GO. The results for *SCN5A* and an overall combination of *in silico* tools for all three genes did not appear promising. However, for *SCN5A* a combination of sequence and evolutionary conservation-based method with a supervised-learning method that uses a wide-spread method may be the best choice. In this study, SNAP appeared to work better than the other five *in silico* tools in analysing *SCN5A* gene variants. SNAP is a supervised-learning method using neural networks to make predictions, and it incorporates evolutionary constraints, structural features and protein annotation information [19].

## Conclusions

**Figure 4:**
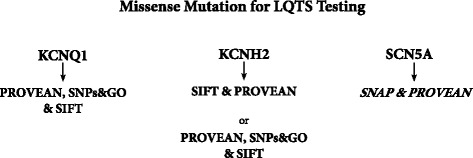
Procedure used for assessing new missense mutations using *in silico* prediction tools for KCNQ1, KCNH2 and SCN5A. The *in silico* prediction tools for SCN5A are italicised as SNAP & PROVEAN should be used with caution for the prediction of *SCN5A* SNVs.

The reliance on *in silico* prediction tools to make a diagnosis is high in light of the inability to functionally characterise each LQTS mutation that is discovered in diagnostic laboratories. This study confirms that these tools must never be relied on as the final arbiter of pathogenicity. Rather, a result should be seen as raising or lowering probability. This study shows that the level of confidence in the result and the combinations of prediction tools with the best performance are gene-dependent, and metaservers, while better than the single *in silico* prediction tools, did not perform better than the combinations of *in silico* tools with the best performance for each of the LQTS genes investigated. The assessment of variants in the *SCN5A* gene is significantly less reliable than those in the *KCNQ1* or *KCNH2* genes*,* and should be used with caution. Figure 4 summarises the *in silico* tools with the best performance for all three genes.

Wherever possible, clinical family-based co-segregation studies centred around clinical registries, and supported with *in vitro* evidence, should form the basis of an assignment of pathogenicity [3,7]. Careful genetic counselling should convey the probabilistic nature of genetic testing both to the patient and their attending clinicians.

## Additional files

Additional file 1:
**Data tables for all the mutations for** ***KCNQ1***, ***KCNH2*** **and** ***SCN5A*** **genes that were analysed and their respective results.**
Additional file 2:
**Figure legends for all five supplementary figures. The zipped file contains all supplementary figures in eps format.**
Additional file 3:
**Raw data.** The zipped file contains all raw data results in txt format from the five different *in silico* prediction tools and two metaservers. Additional file 4:
**Tables S1-S4.** Data tables of the pairwise correlation of *in silico* prediction tools for ***KCNQ1***, ***KCNH2*** and ***SCN5A*** genes, and all three genes.

## Abbreviations

BrS: Brugada syndrome
ECG: Electrocardiogram
FP: False positives
FN: False negatives
GO: Gene ontology
*KCNQ1* gene: Potassium voltage-gated channel, KQT-like subfamily, member 1(LQT1)
*KCNH2* gene: Potassium voltage-gated channel, subfamily H (eag-related), member 2 (LQT2)
LOVD: Leiden open variation database
LQTS: Long QT syndrome
MCC: Matthews correlation coefficient
PROVEAN: Protein variation effect analyzer
*SCN5A* gene: sodium channel, voltage-gated, type V, alpha subunit (LQT3)
SNVs: Single nucleotide variants
TP: True positives
TN: True negatives

## References

[1] Chung SK, MacCormick JM, McCulley CH, Crawford J, Eddy CA, Mitchell EA (2007). Long QT and Brugada syndrome gene mutations in New Zealand. Heart Rhythm.

[2] Schwartz PJ, Stramba-Badiale M, Crotti L, Pedrazzini M, Besana A, Bosi G (2009). Prevalence of the congenital long-QT syndrome. Circulation.

[3] Leong IU, Skinner J, Love D (2014). Application of massively parallel sequencing in the clinical diagnostic testing of inherited cardiac conditions. Med Sci.

[4] Splawski I, Shen J, Timothy KW, Lehmann MH, Priori S, Robinson JL (2000). Spectrum of mutations in long-QT syndrome genes. KVLQT1, HERG, SCN5A, KCNE1, and KCNE2. Circulation.

[5] Giudicessi JR, Kapplinger JD, Tester DJ, Alders M, Salisbury BA, Wilde AA (2012). Phylogenetic and physicochemical analyses enhance the classification of rare nonsynonymous single nucleotide variants in type 1 and 2 long-QT syndrome. Circ Cardiovasc Genet.

[6] Shimizu W (2014). Clinical and genetic diagnosis for inherited cardiac arrhythmias. J Nippon Med Sch.

[7] Earle N, Crawford J, Smith W, Hayes I, Shelling A, Hood M (2013). Community detection of long QT syndrome with a clinical registry: an alternative to ECG screening programs?. Heart Rhythm.

[8] Kapplinger JD, Tester DJ, Salisbury BA, Carr JL, Harris-Kerr C, Pollevick GD (2009). Spectrum and prevalence of mutations from the first 2,500 consecutive unrelated patients referred for the FAMILION long QT syndrome genetic test. Heart Rhythm.

[9] Ackerman MJ, Splawski I, Makielski JC, Tester DJ, Will ML, Timothy KW (2004). Spectrum and prevalence of cardiac sodium channel variants among black, white, Asian, and Hispanic individuals: implications for arrhythmogenic susceptibility and Brugada/long QT syndrome genetic testing. Heart Rhythm.

[10] Ackerman MJ, Tester DJ, Jones GS, Will ML, Burrow CR, Curran ME (2003). Ethnic differences in cardiac potassium channel variants: implications for genetic susceptibility to sudden cardiac death and genetic testing for congenital long QT syndrome. Mayo Clin Proc.

[11] Tavtigian SV, Greenblatt MS, Lesueur F, Byrnes GB (2008). In silico analysis of missense substitutions using sequence-alignment based methods. Hum Mutat.

[12] Bioinformatic tool and resource analysis. [http://www.ngrl.org.uk/Manchester/projects/bioinformatic-tools]

[13] Hou J, Ma J, Shen B (2013). Identifying driver mutations in cancer. Bioinformatic for diagnosis, prognosis and treatment of complex diseases.

[14] Ng PC, Henikoff S (2001). Predicting deleterious amino acid substitutions. Genome Res.

[15] Ng PC, Henikoff S (2002). Accounting for human polymorphisms predicted to affect protein function. Genome Res.

[16] Choi Y, Sims GE, Murphy S, Miller JR, Chan AP (2012). Predicting the functional effect of amino acid substitutions and indels. PLoS One.

[17] Adzhubei IA, Schmidt S, Peshkin L, Ramensky VE, Gerasimova A, Bork P (2010). A method and server for predicting damaging missense mutations. Nat Methods.

[18] Calabrese R, Capriotti E, Fariselli P, Martelli PL, Casadio R (2009). Functional annotations improve the predictive score of human disease-related mutations in proteins. Hum Mutat.

[19] Bromberg Y, Rost B (2007). SNAP: predict effect of non-synonymous polymorphisms on function. Nucleic Acids Res.

[20] Chan PA, Duraisamy S, Miller PJ, Newell JA, McBride C, Bond JP (2007). Interpreting missense variants: comparing computational methods in human disease genes CDKN2A, MLH1, MSH2, MECP2, and tyrosinase (TYR). Hum Mutat.

[21] Chun S, Fay JC (2009). Identification of deleterious mutations within three human genomes. Genome Res.

[22] Flanagan SE, Patch AM, Ellard S (2010). Using SIFT and PolyPhen to predict loss-of-function and gain-of-function mutations. Genet Test Mol Biomarkers.

[23] Hicks S, Wheeler DA, Plon SE, Kimmel M (2011). Prediction of missense mutation functionality depends on both the algorithm and sequence alignment employed. Hum Mutat.

[24] Balasubramanian S, Xia Y, Freinkman E, Gerstein M (2005). Sequence variation in G-protein-coupled receptors: analysis of single nucleotide polymorphisms. Nucleic Acids Res.

[25] Mathe E, Olivier M, Kato S, Ishioka C, Hainaut P, Tavtigian SV (2006). Computational approaches for predicting the biological effect of p53 missense mutations: a comparison of three sequence analysis based methods. Nucleic Acids Res.

[26] Bao L, Cui Y (2005). Prediction of the phenotypic effects of non-synonymous single nucleotide polymorphisms using structural and evolutionary information. Bioinformatics (Oxford, England).

[27] Chao EC, Velasquez JL, Witherspoon MS, Rozek LS, Peel D, Ng P (2008). Accurate classification of MLH1/MSH2 missense variants with multivariate analysis of protein polymorphisms-mismatch repair (MAPP-MMR). Hum Mutat.

[28] Karchin R (2009). Next generation tools for the annotation of human SNPs. Brief Bioinform.

[29] Olatubosun A, Valiaho J, Harkonen J, Thusberg J, Vihinen M (2012). PON-P: integrated predictor for pathogenicity of missense variants. Hum Mutat.

[30] Capriotti E, Altman RB, Bromberg Y (2013). Collective judgment predicts disease-associated single nucleotide variants. BMC Genomics.

[31] Bendl J, Stourac J, Salanda O, Pavelka A, Wieben ED, Zendulka J (2014). PredictSNP: robust and accurate consensus classifier for prediction of disease-related mutations. PLoS Comput Biol.

[32] Napolitano C, Wilson J, deGiuli L. Inherited arrhythmias database. In: Pavia, Italy and New York, USA: IRCCS Fondazione Salvatore Maugeri and Cardiovascular Genetics Program; 2000: 1.

[33] Kapplinger JD, Tester DJ, Alders M, Benito B, Berthet M, Brugada J (2010). An international compendium of mutations in the SCN5A-encoded cardiac sodium channel in patients referred for Brugada syndrome genetic testing. Heart Rhythm.

[34] Zhang T, Moss A, Cong P, Pan M, Chang B, Zheng L (2010). LQTS gene LOVD database. Hum Mutat.

[35] Capriotti E, Calabrese R, Fariselli P, Martelli PL, Altman RB, Casadio R (2013). WS-SNPs&GO: a web server for predicting the deleterious effect of human protein variants using functional annotation. BeMC Genomics.

[36] Baldi P, Brunak S, Chauvin Y, Andersen CA, Nielsen H (2000). Assessing the accuracy of prediction algorithms for classification: an overview. Bioinformatics (Oxford, England).

[37] Matthews BW (1975). Comparison of the predicted and observed secondary structure of T4 phage lysozyme. Biochim Biophys Acta.

[38] Vihinen M (2012). How to evaluate performance of prediction methods? Measures and their interpretation in variation effect analysis. BMC Genomics.

[39] Fawcett T (2006). An introduction to ROC analysis. Pattern Recogn Lett.

[40] Robin X, Turck N, Hainard A, Tiberti N, Lisacek F, Sanchez JC (2011). pROC: an open-source package for R and S+ to analyze and compare ROC curves. BMC Bioinformatics.

[41] Kruskal WH, Wallis WA (1952). Use of ranks in one-criterion variance analysis. J Am Stat Assoc.

[42] Tester DJ, Ackerman MJ (2008). Novel gene and mutation discovery in congenital long QT syndrome: let's keep looking where the street lamp standeth. Heart Rhythm.

[43] Bezzina CR, Rook MB, Wilde AA (2001). Cardiac sodium channel and inherited arrhythmia syndromes. Cardiovasc Res.

[44] Remme CA, Wilde AA (2008). SCN5A overlap syndromes: no end to disease complexity?. Europace.

[45] Rivolta I, Abriel H, Tateyama M, Liu H, Memmi M, Vardas P (2001). Inherited Brugada and long QT-3 syndrome mutations of a single residue of the cardiac sodium channel confer distinct channel and clinical phenotypes. J Biol Chem.

[46] Priori SG, Napolitano C, Schwartz PJ, Bloise R, Crotti L, Ronchetti E (2000). The elusive link between LQT3 and Brugada syndrome: the role of flecainide challenge. Circulation.

[47] Gnad F, Baucom A, Mukhyala K, Manning G, Zhang Z (2013). Assessment of computational methods for predicting the effects of missense mutations in human cancers. BMC genomics.

[48] Makielski JC, Ye B, Valdivia CR, Pagel MD, Pu J, Tester DJ (2003). A ubiquitous splice variant and a common polymorphism affect heterologous expression of recombinant human SCN5A heart sodium channels. Circ Res.

[49] Tan BH, Valdivia CR, Rok BA, Ye B, Ruwaldt KM, Tester DJ (2005). Common human SCN5A polymorphisms have altered electrophysiology when expressed in Q1077 splice variants. Heart Rhythm.

[50] Borchert B, Lawrenz T, Stellbrink C (2006). Long and short QT syndrome. Herzschrittmacherther Elektrophysiol.

