# Supplementary material for: Assessment of the predictive accuracy of five in silico prediction tools, alone or in combination, and two metaservers to classify long QT syndrome gene mutations
Source: BMC Med Genet. 2015 May 13;16:34. doi: 10.1186/s12881-015-0176-z (PMC4630850; doi:10.1186/s12881-015-0176-z)
Supplement: Additional file 2: — Figure legends for all five supplementary figures. The zipped file contains all supplementary figures in eps format. [file 12881_2015_176_MOESM2_ESM.zip › 9367791921483797_add4.pdf]

## Supplementary Figure Legends

### Supplementary Figure 1

Amino acid sequence of KCNQ1 (LQTS 1) with the locations of transmembrane domains (blue arrows), pore helix/selection filter (yellow/orange arrows, respectively), helix A – D (pink rods), benign SNVs (green) and pathogenic SNVs (red). The figure was produced by Geneious vR6.1.6 [1].

### Supplementary Figure 2

Amino acid sequence of KCNH2 (LQTS 2) with the locations of the EAG domain (green arrow), Cap region (pink arrow), PAS domain (pink arrow), transmembrane domains (blue arrows), pore helix (yellow), C-linker/cNBD (green arrow), CNB domain (pink arrow), benign SNVs (green) and pathogenic SNVs (red). The figure was produced by Geneious vR6.1.6 [1].

### Supplementary Figure 3

Amino acid sequence of SCN5A (LQTS 3) with the locations of the transmembrane domains (blue arrows), pore regions (yellow), benign SNVs (green) and pathogenic SNVs (red). The figure was produced by Geneious vR6.1.6 [1].

### Supplemntary Figure 4

Predictions of pathogenic and benign SNVs with SIFT, PolyPhen-2, PROVEAN, SNPs&GO and SNAP for *KCNQ1* and *KCNH2*. The overlap between all five *in silico* tools (yellow) represents SNVs with the same predictions. The orange regions highlight the additional overlap between PROVEAN, SNPs&GO and SIFT.

**Supplementary Figure 5**

Predictions of pathogenic and benign SNVs with SIFT, PolyPhen-2, PROVEAN, SNPs&GO and SNAP for *SCN5A* and all genes. The overlap between all five *in silico* tools (yellow) represents SNVs with the same predictions. The orange regions highlight the additional overlap between SNAP and PROVEAN (*SCN5A*) and SIFT, PROVEAN and SNAP (all genes)

**Reference**

[1] Biomatters. Genegene vr6.1.6.
