# Supplementary material for: Assessment of the predictive accuracy of five in silico prediction tools, alone or in combination, and two metaservers to classify long QT syndrome gene mutations
Source: BMC Med Genet. 2015 May 13;16:34. doi: 10.1186/s12881-015-0176-z (PMC4630850; doi:10.1186/s12881-015-0176-z)
Supplement: Additional file 4: — Tables S1-S4. Data tables of the pairwise correlation of in silico prediction tools for KCNQ1, KCNH2 and SCN5A genes, and all three genes. [file 12881_2015_176_MOESM4_ESM.docx]

**Supplementary Table 1:** Pairwise correlation of *in silico* prediction tools for *KCNQ1*

|  | **PROVEAN** | **PolyPhen-2** | **SIFT** | **SNPs&GO** |
| --- | --- | --- | --- | --- |
| **PolyPhen-2** | 0.439 |  |  |  |
| **SIFT** | 0.610 | 0.427 |  |  |
| **SNPs&GO** | 0.035 | 0.395 | 0.074 |  |
| **SNAP** | 0.411 | 0.503 | 0.202 | 0.405 |

**Supplementary Table 2:** Pairwise correlation of *in silico* prediction tools for *KCNH2*

|  | **PROVEAN** | **PolyPhen-2** | **SIFT** | **SNPs&GO** |
| --- | --- | --- | --- | --- |
| **PolyPhen-2** | 0.442 |  |  |  |
| **SIFT** | 0.601 | 0.298 |  |  |
| **SNPs&GO** | 0.300 | 0.322 | 0.197 |  |
| **SNAP** | 0.575 | 0.421 | 0.572 | 0.448 |

**Supplementary Table 3:** Pairwise correlation of *in silico* prediction tools for *SCN5A*

|  | **PROVEAN** | **PolyPhen-2** | **SIFT** | **SNPs&GO** |
| --- | --- | --- | --- | --- |
| **PolyPhen-2** | 0.547 |  |  |  |
| **SIFT** | 0.590 | 0.564 |  |  |
| **SNPs&GO** | 0.389 | 0.403 | 0.403 |  |
| **SNAP** | 0.580 | 0.429 | 0.429 | 0.452 |

**Supplementary Table 4:** Pairwise correlation of *in silico* prediction tools for all genes

|  | **PROVEAN** | **PolyPhen-2** | **SIFT** | **SNPs&GO** |
| --- | --- | --- | --- | --- |
| **PolyPhen-2** | 0.506 |  |  |  |
| **SIFT** | 0.606 | 0.421 |  |  |
| **SNPs&GO** | 0.441 | 0.496 | 0.354 |  |
| **SNAP** | 0.437 | 0.423 | 0.416 | 0.403 |
