# Supplementary material for: Assessment of the predictive accuracy of five in silico prediction tools, alone or in combination, and two metaservers to classify long QT syndrome gene mutations
Source: BMC Med Genet. 2015 May 13;16:34. doi: 10.1186/s12881-015-0176-z (PMC4630850; doi:10.1186/s12881-015-0176-z)
Supplement: Additional file 2: — Figure legends for all five supplementary figures. The zipped file contains all supplementary figures in eps format. [file 12881_2015_176_MOESM2_ESM.zip › 9367791921483797_add5.zip!/Suppl files pdf/Suppl Fig 1.pdf]

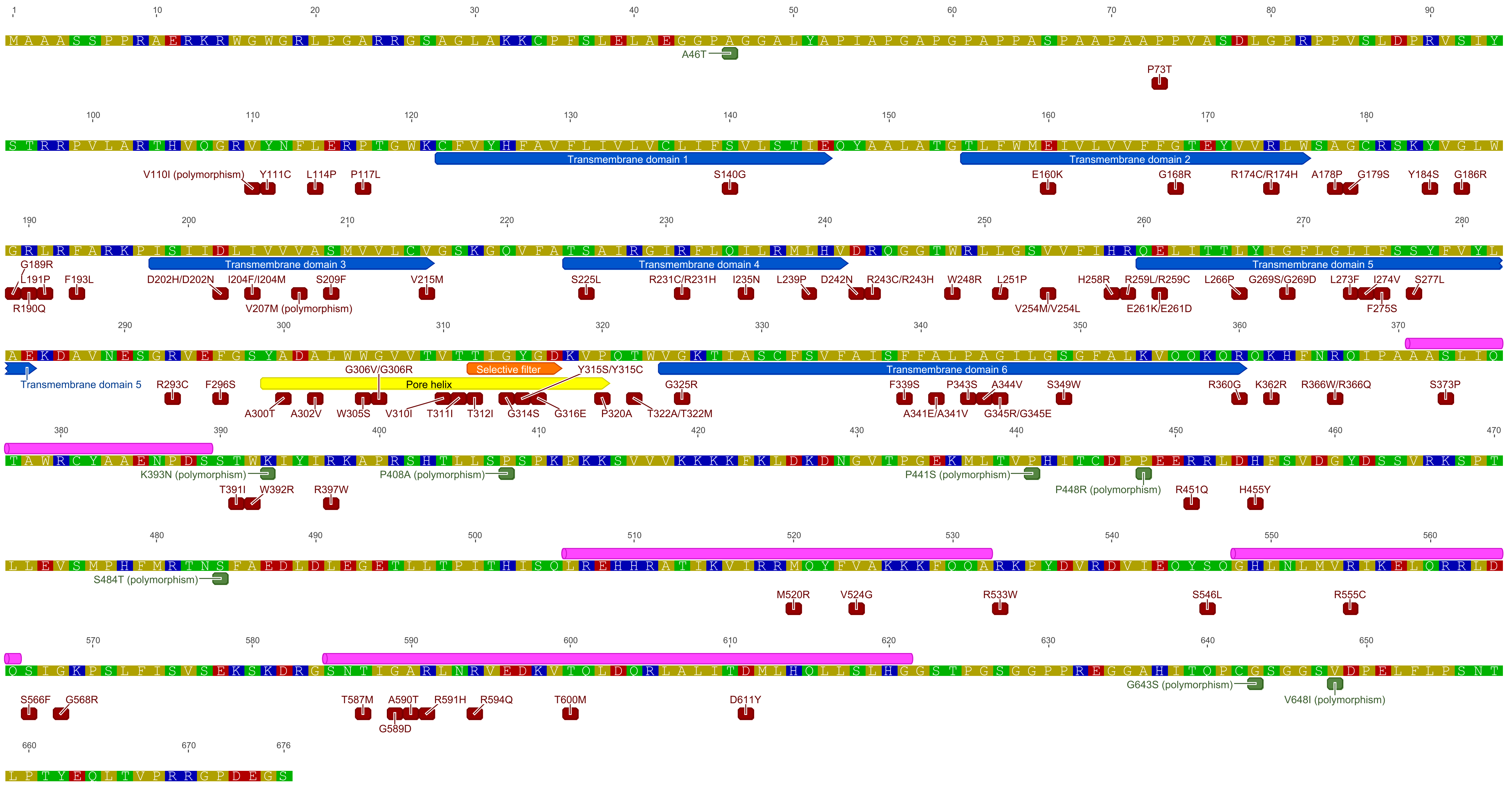

A46T
P73T
V110I (polymorphism)
Y111C
L114P
P117L
Transmembrane domain 1
S140G
Transmembrane domain 2
E160K
G168R
R174C/R174H
A178P
G179S
Y184S
G186R
G189R
L191P
F193L
R190Q
Transmembrane domain 3
D202H/D202N
I204F/I204M
S209F
V207M (polymorphism)
V215M
Transmembrane domain 4
S225L
R231C/R231H
I235N
L239P
D242N
R243C/R243H
W248R
L251P
V254M/V254L
H258R
R259L/R259C
E261K/E261D
L266P
G269S/G269D
Transmembrane domain 5
L273F
I274V
S277L
F275S
Transmembrane domain 5
R293C
F296S
G306V/G306R
Selective filter
Y315S/Y315C
Pore helix
A300T
A302V
W305S
V310I
T311I
T312I
G314S
G316E
P320A
T322A/T322M
Transmembrane domain 6
G325R
F339S
P343S
A344V
S349W
A341E/A341V
G345R/G345E
R360G
K362R
R366W/R366Q
S373P
K393N (polymorphism)
P408A (polymorphism)
P441S (polymorphism)
P448R (polymorphism)
T391I
W392R
R397W
R451Q
H455Y
S484T (polymorphism)
M520R
V524G
R533W
S546L
R555C
G643S (polymorphism)
V648I (polymorphism)
S566F
G568R
T587M
A590T
R591H
R594Q
T600M
D611Y
G589D
