# Supplementary material for: Assessment of the predictive accuracy of five in silico prediction tools, alone or in combination, and two metaservers to classify long QT syndrome gene mutations
Source: BMC Med Genet. 2015 May 13;16:34. doi: 10.1186/s12881-015-0176-z (PMC4630850; doi:10.1186/s12881-015-0176-z)
Supplement: Additional file 2: — Figure legends for all five supplementary figures. The zipped file contains all supplementary figures in eps format. [file 12881_2015_176_MOESM2_ESM.zip › 9367791921483797_add5.zip!/Suppl files pdf/Suppl Fig 2.pdf]

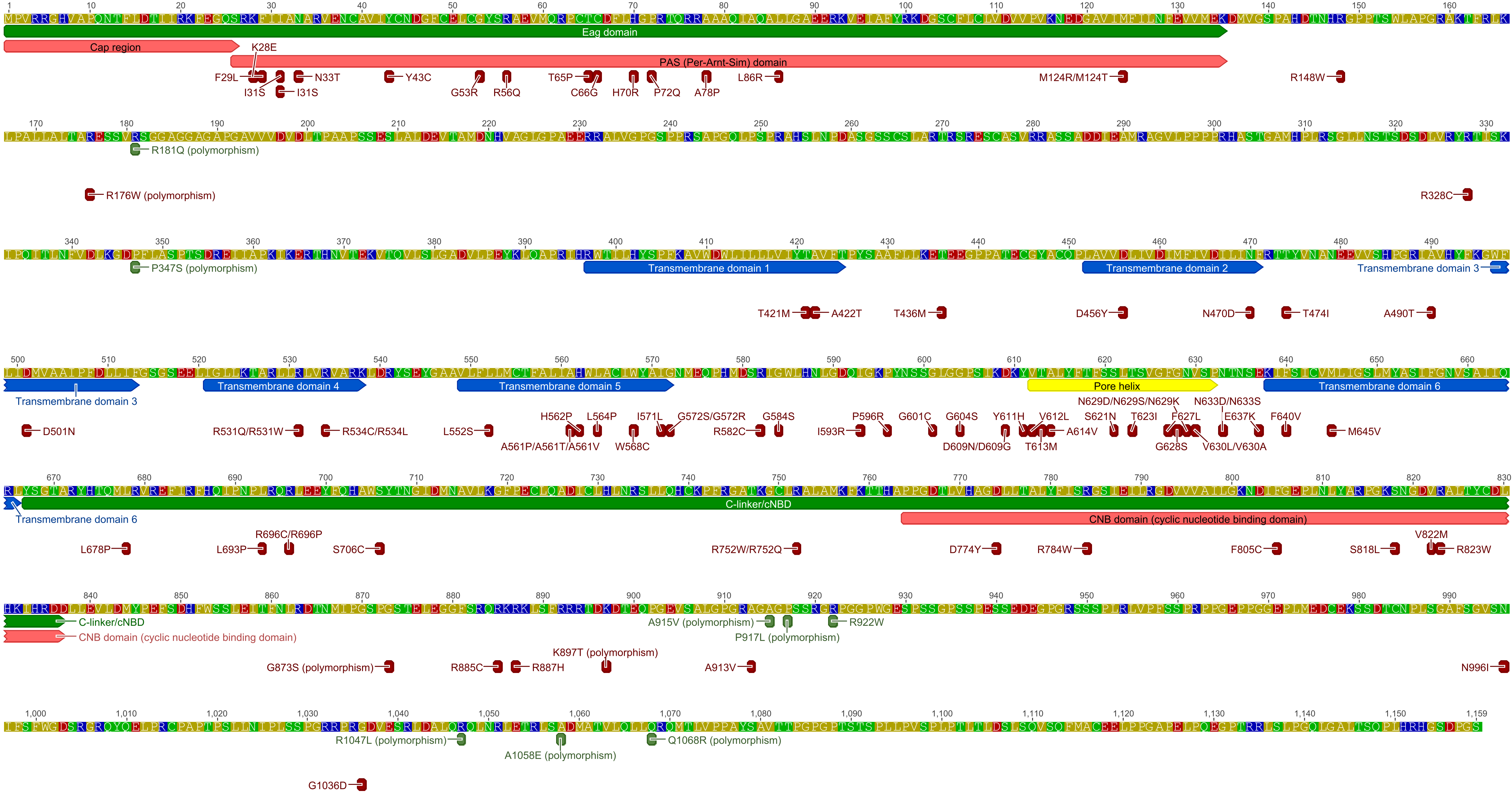

Eag domain
Cap region
K28E
PAS (Per-Arnt-Sim) domain
F29L
I31S
I31S
N33T
Y43C
G53R
R56Q
T65P
C66G
H70R
P72Q
A78P
L86R
M124R/M124T
R148W
R181Q (polymorphism)
R176W (polymorphism)
R328C
P347S (polymorphism)
Transmembrane domain 1
Transmembrane domain 2
Transmembrane domain 3
T421M
A422T
T436M
D456Y
N470D
T474I
A490T
Transmembrane domain 3
Transmembrane domain 4
Transmembrane domain 5
Pore helix
Transmembrane domain 6
N629D/N629S/N629K
N633D/N633S
H562P
L564P
I571L
G572S/G572R
G584S
P596R
G601C
G604S
Y611H
V612L
S621N
T623I
F627L
E637K
F640V
D501N
R531Q/R531W
R534C/R534L
L552S
R582C
I593R
A614V
M645V
A561P/A561T/A561V
W568C
D609N/D609G
T613M
G628S
V630L/V630A
C-linker/cNBD
Transmembrane domain 6
CNB domain (cyclic nucleotide binding domain)
R696C/R696P
V822M
L678P
L693P
S706C
R752W/R752Q
D774Y
R784W
F805C
S818L
R823W
C-linker/cNBD
CNB domain (cyclic nucleotide binding domain)
A915V (polymorphism)
P917L (polymorphism)
R922W
K897T (polymorphism)
G873S (polymorphism)
R885C
R887H
A913V
N996I
R1047L (polymorphism)
A1058E (polymorphism)
Q1068R (polymorphism)
G1036D
