# Supplementary material for: Assessment of the predictive accuracy of five in silico prediction tools, alone or in combination, and two metaservers to classify long QT syndrome gene mutations
Source: BMC Med Genet. 2015 May 13;16:34. doi: 10.1186/s12881-015-0176-z (PMC4630850; doi:10.1186/s12881-015-0176-z)
Supplement: Additional file 2: — Figure legends for all five supplementary figures. The zipped file contains all supplementary figures in eps format. [file 12881_2015_176_MOESM2_ESM.zip › 9367791921483797_add5.zip!/Suppl files pdf/Suppl Fig 3.pdf]

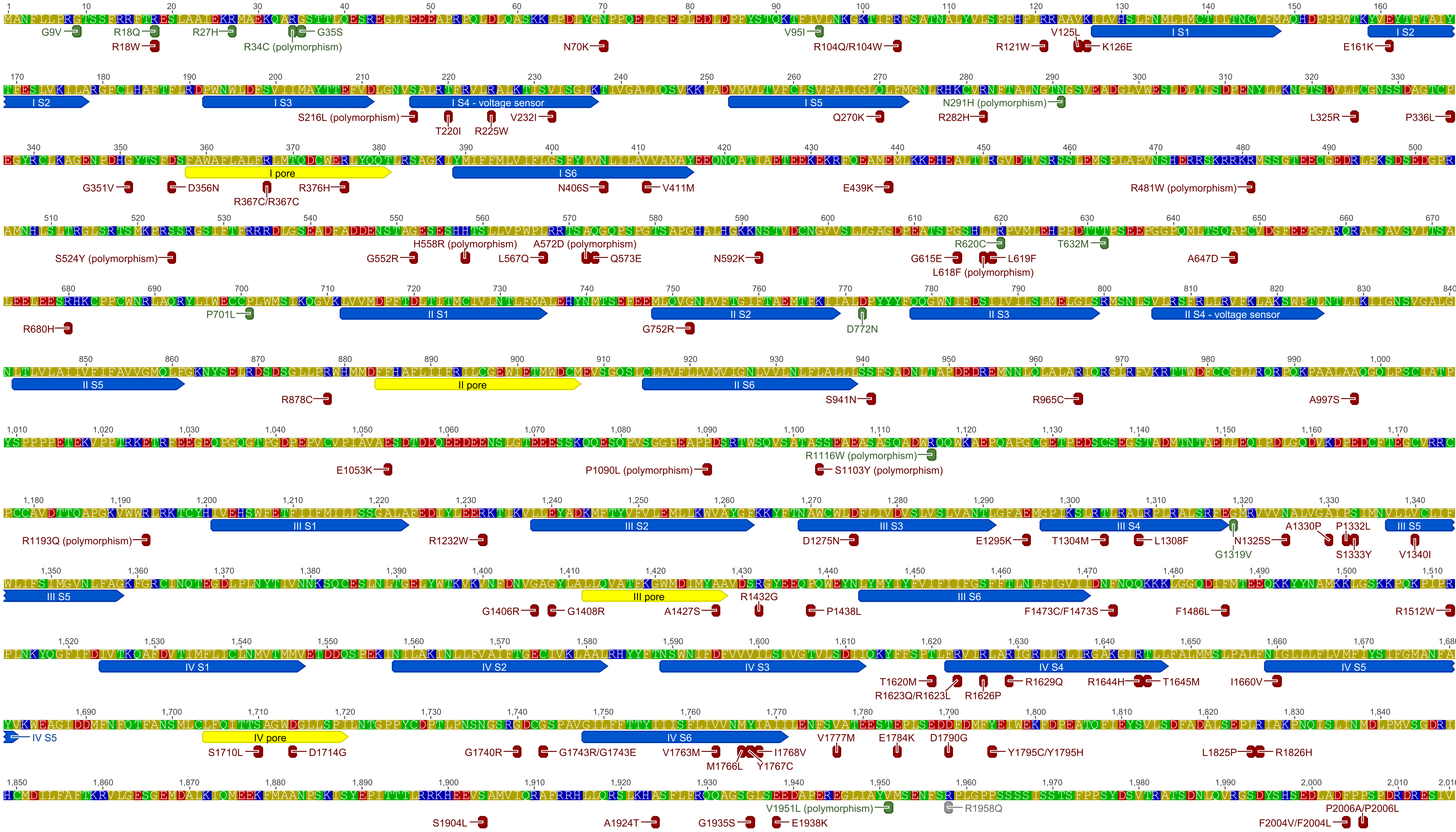
G9V
R18Q
R18W
R27H
G35S
R34C (polymorphism)
N70K
V95I
R104Q/R104W
R121W
V125L
K126E
I S1
I S2
E161K
I S3
I S4 - voltage sensor
I S5
S216L (polymorphism)
T220I
R225W
V232I
Q270K
R282H
N291H (polymorphism)
L325R
P336L
I pore
I S6
G351V
D356N
R367C/R367C
R376H
N406S
V411M
E439K
R481W (polymorphism)
H558R (polymorphism)
A572D (polymorphism)
S524Y (polymorphism)
G552R
L567Q
Q573E
N592K
R620C
T632M
G615E
L619F
L618F (polymorphism)
A647D
P701L
II S1
II S2
II S3
II S4 - voltage sensor
R680H
G752R
D772N
II S5
II pore
II S6
R878C
S941N
R965C
A997S
R1116W (polymorphism)
E1053K
P1090L (polymorphism)
S1103Y (polymorphism)
III S1
III S2
III S3
III S4
III S5
R1193Q (polymorphism)
R1232W
D1275N
E1295K
T1304M
L1308F
N1325S
G1319V
A1330P
P1332L
S1333Y
V1340I
III pore
III S6
G1406R
G1408R
A1427S
R1432G
P1438L
F1473C/F1473S
F1486L
R1512W
IV S1
IV S2
IV S3
IV S4
IV S5
T1620M
R1623Q/R1623L
R1626P
R1629Q
R1644H
T1645M
I1660V
IV pore
IV S6
S1710L
D1714G
G1740R
G1743R/G1743E
V1763M
M1766L
Y1767C
I1768V
V1777M
E1784K
D1790G
Y1795C/Y1795H
L1825P
R1826H
V1951L (polymorphism)
R1958Q
S1904L
A1924T
G1935S
E1938K
P2006A/P2006L
F2004V/F2004L
