# Supplementary material for: Assessment of the predictive accuracy of five in silico prediction tools, alone or in combination, and two metaservers to classify long QT syndrome gene mutations
Source: BMC Med Genet. 2015 May 13;16:34. doi: 10.1186/s12881-015-0176-z (PMC4630850; doi:10.1186/s12881-015-0176-z)
Supplement: Additional file 2: — Figure legends for all five supplementary figures. The zipped file contains all supplementary figures in eps format. [file 12881_2015_176_MOESM2_ESM.zip › 9367791921483797_add5.zip!/Suppl files pdf/Suppl Fig 4.pdf]

KCNQ1

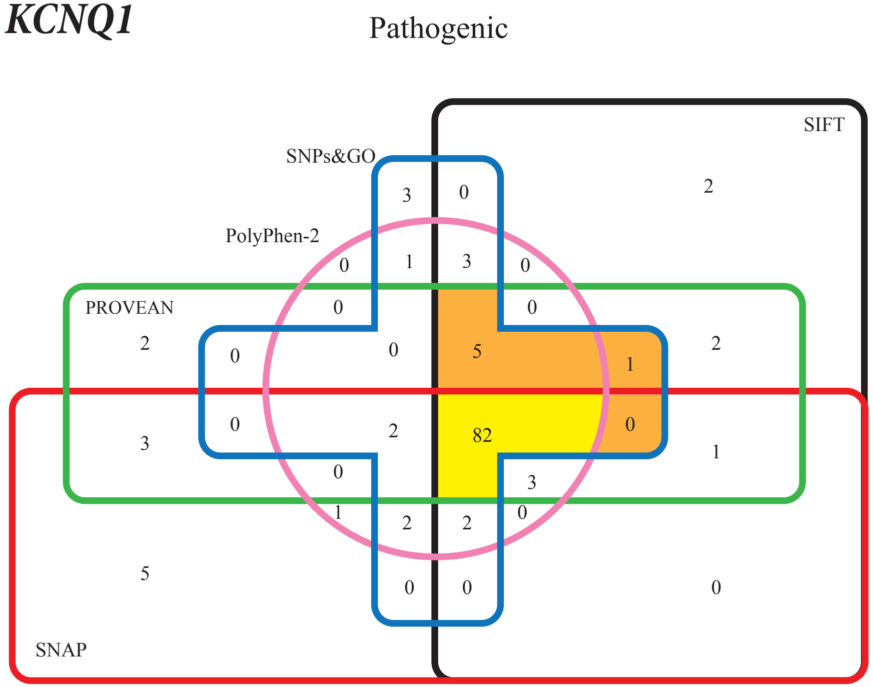
Pathogenic
SIFT
SNPs&GO
PolyPhen-2
PROVEAN
SNAP
2
3
0
0
1
3
0
0
0
0
5
1
2
2
0
0
2
82
0
3
1
3
0
0
1
2
2
0
5
0
0
0

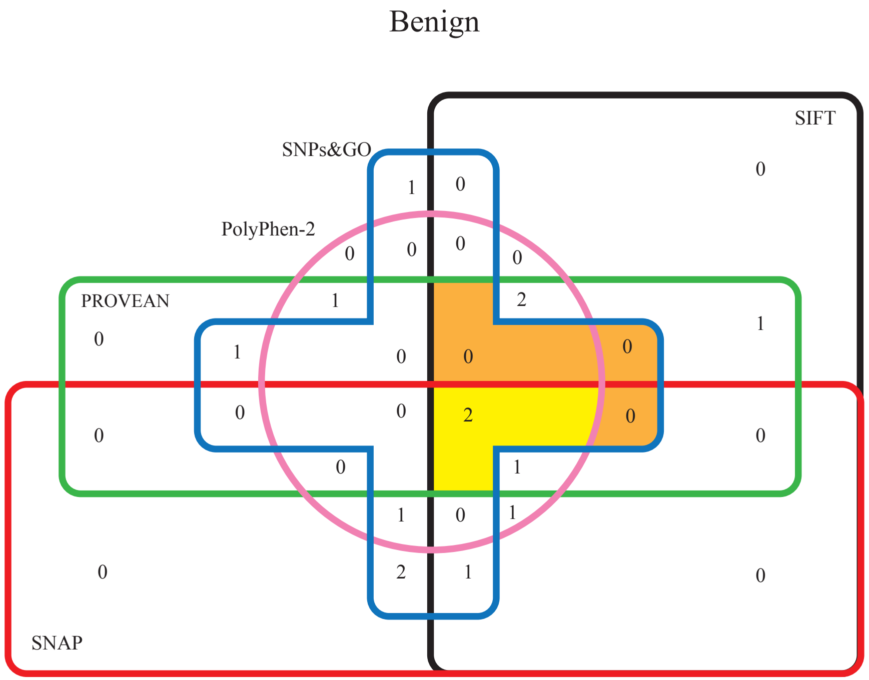
Benign
SIFT
SNPs&GO
PolyPhen-2
PROVEAN
SNAP
0
1
0
0
0
0
0
1
2
0
1
0
0
0
1
0
0
0
2
0
0
0
1
0
1
1
0
1
0
2
1
0

KCNH2

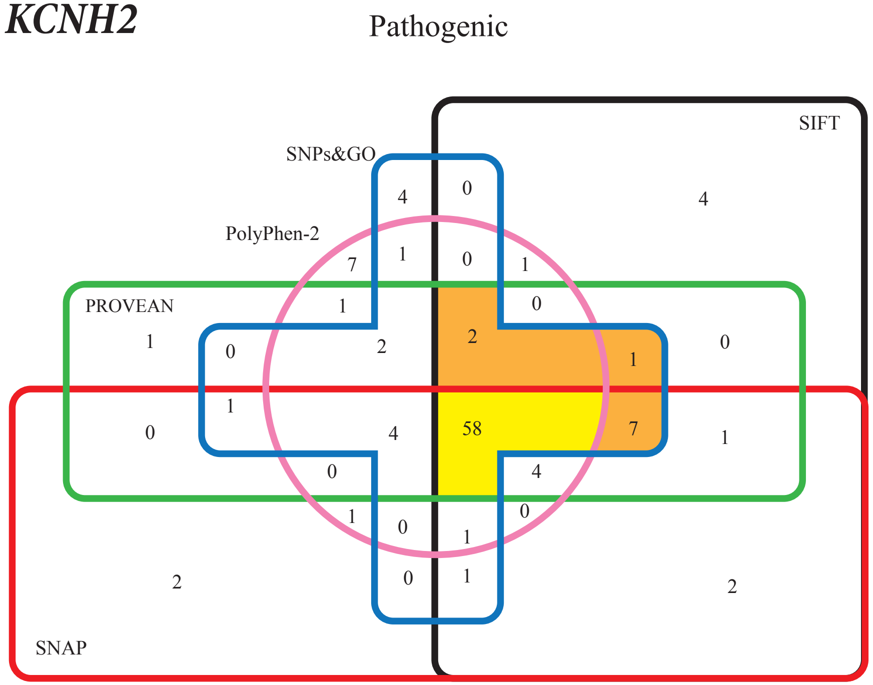
Pathogenic
SIFT
SNPs&GO
PolyPhen-2
PROVEAN
SNAP
4
0
4
7
1
0
1
1
0
1
0
2
2
1
0
0
1
4
58
7
1
0
4
1
0
0
1
0
2
0
1
2

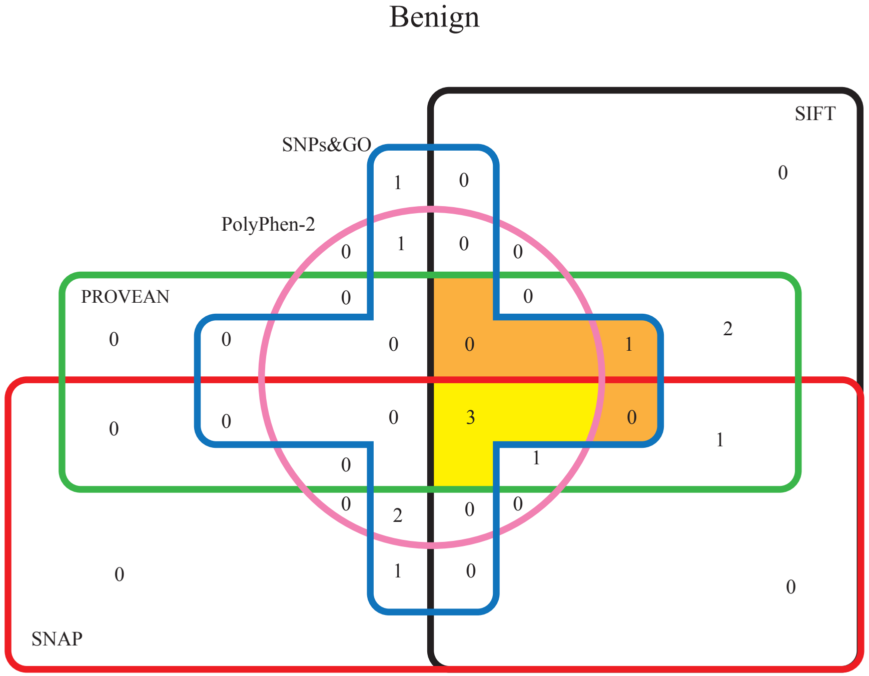
Benign
SIFT
SNPs&GO
PolyPhen-2
PROVEAN
SNAP
1
0
0
0
1
0
0
0
0
0
0
0
0
1
2
0
0
0
3
0
1
0
1
0
0
2
0
0
0
1
0
0
