# Supplementary material for: Assessment of the predictive accuracy of five in silico prediction tools, alone or in combination, and two metaservers to classify long QT syndrome gene mutations
Source: BMC Med Genet. 2015 May 13;16:34. doi: 10.1186/s12881-015-0176-z (PMC4630850; doi:10.1186/s12881-015-0176-z)

SCN5A

Pathogenic

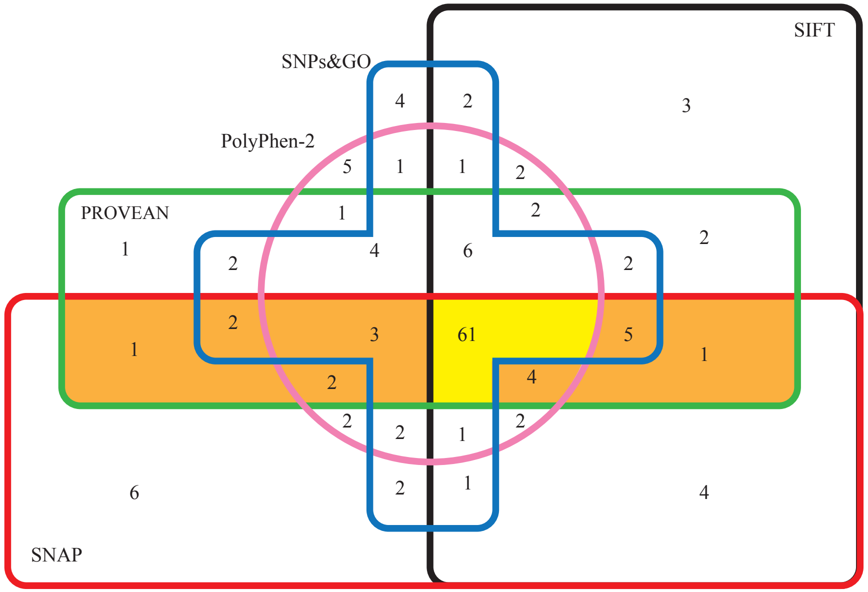

Benign

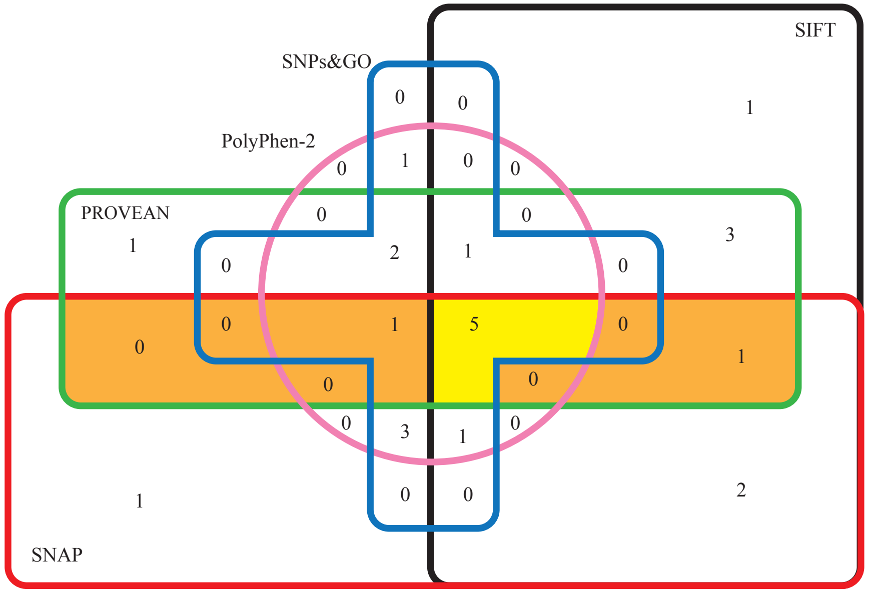

All genes

Pathogenic

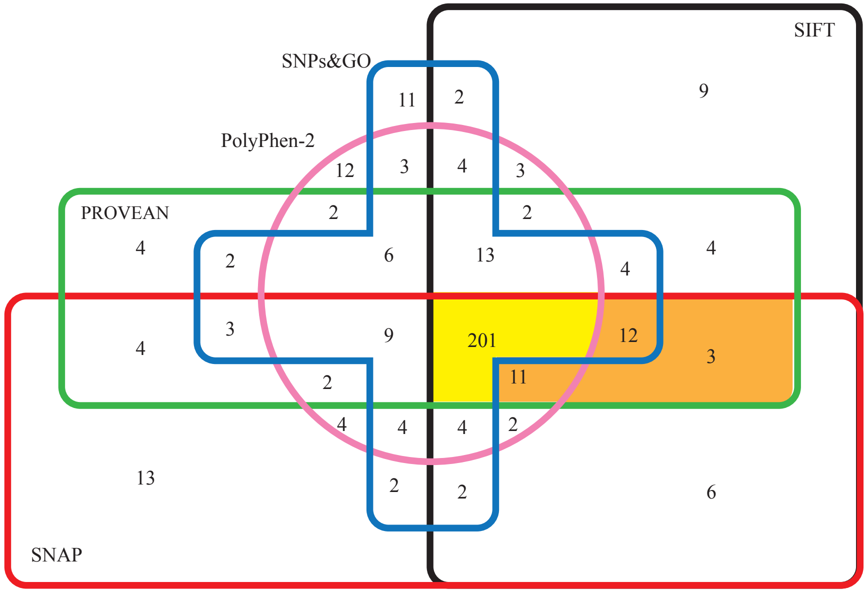

Benign

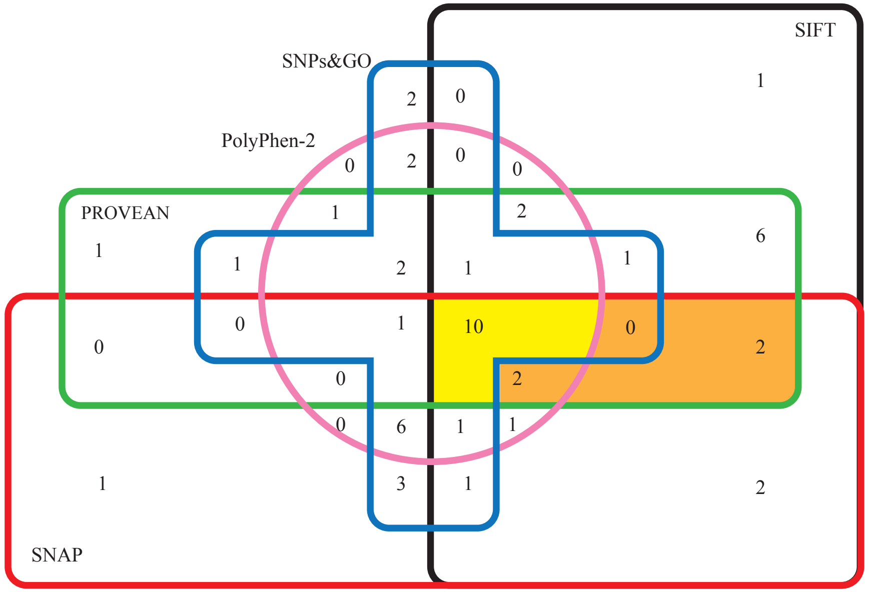

Supplement: Additional file 2: — Figure legends for all five supplementary figures. The zipped file contains all supplementary figures in eps format. [file 12881_2015_176_MOESM2_ESM.zip › 9367791921483797_add5.zip!/Suppl files pdf/Suppl Fig 5.pdf]
